# Anxiety disorders and intravenous drug use in chemsex context

**DOI:** 10.1192/j.eurpsy.2024.730

**Published:** 2024-08-27

**Authors:** J. Curto Ramos, A. Rodríguez Laguna, P. Barrio, L. Ibarguchi, A. García, I. Azqueta, H. Dolengevich Segal

**Affiliations:** ^1^Department of Psychiatry, Clinical Psychology and Mental Health, La Paz University Hospital; ^2^ Apoyo Positivo; ^3^Dual Disorders Program. Department of Psychiatry, Henares University Hospital, Madrid, Spain

## Abstract

**Introduction:**

Several studies have called atention to the mental health disorders associated with chemsex -the intentional use of drugs before or during sexual intercourse GBMSM (gay, bisexual and men who have sex with men) population-. Sexualized intravenous drug use is also known as slam or slamsex. There are few studies that analyze the mental health differences between intravenous drug users compared to non-intravenous drug users in chemsex context.

**Objectives:**

We aim to describe the mental health outcomes including current and past anxiety disorders diagnosis in a sample of users with sexualized drug use (chemsex) attended by the non-governmental organization Apoyo Positivo in the program “Sex, Drugs and You” and to compare the differences of current and previous diagnosis of anxiety disorders between intravenous drug users compared to non-intravenous drug users.

**Methods:**

A cross-sectional descriptive analysis of a sample of users attended by the non-governmental organization Apoyo Positivo in the program “Sex, Drugs and You” between 2016-2019 was performed.

**Results:**

We included 217 participants. Current or past diagnosis of anxiety disorders was found in 142 participants. Anxiety disorders were significantly higher in the intravenous drug use group compared to the non-intravenous drug use group (p<0.05).

**Conclusions:**

Previous studies have reported that MSM who practiced chemsex were more likely to experience from anxiety. In our study, anxiety disorders where higher in participantes who engaged in intravenous drug use. A multidisciplinary team is necessary to address chemsex and provide care and treatment for mental health problems such as anxiety, depression, suicidal behaviour or drug-induced psychosis.

**Disclosure of Interest:**

None Declared

